# Childhood survivors of high‐risk neuroblastoma show signs of immune recovery and not immunosenescence

**DOI:** 10.1002/eji.202048541

**Published:** 2020-08-18

**Authors:** Petra Lázničková, Tomáš Kepák, Marcela Hortová – Kohoutková, Luděk Horváth, Kateřina Sheardová, Rafal Marciniak, Carmine Vacca, Michaela Šiklová, Teresa Zelante, Lenka Rossmeislová, Zdenka Křenová, Jaroslav Štěrba, Kamila Bendíčková, Jan Frič

**Affiliations:** ^1^ International Clinical Research Center St. Anne's University Hospital Brno Brno Czech Republic; ^2^ Department of Biology, Faculty of Medicine Masaryk University Brno Czech Republic; ^3^ Department of Paediatric Oncology University Hospital Brno Brno Czech Republic; ^4^ 1st Neurology Department St. Anne's University Hospital Brno Brno Czech Republic; ^5^ Department of Experimental Medicine University of Perugia Perugia Italy; ^6^ Department of Pathophysiology, Third Faculty of Medicine Charles University Prague Czech Republic; ^7^ Institute of Hematology and Blood Transfusion Prague Czech Republic

**Keywords:** adverse late effects, childhood, immune recovery, immunosenescence, neuroblastoma

## Abstract

Neuroblastoma survivors show signs of immunosenescence early after therapy in CD8^+^ T cell compartment and elevated plasma TNF‐α but in later follow‐up immune recovery comes into play. Whether the recovery phenotype is long lasting or transient remains to be elucidated, however, late adverse effects often occur in childhood cancer survivors.

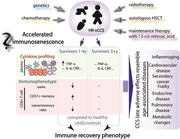

Neuroblastoma is a cancer that develops from immature nerve cells and can arise in multiple areas of the body, most commonly in early childhood. Children diagnosed with high‐risk neuroblastoma undergo intensive treatment potentially including radiotherapy, surgery, chemotherapy, targeted biologic therapy, and autologous hematopoietic stem cell transplantation. As well as aggressively attacking the cancer, this regime also inflicts system‐wide damage at the tissue and cellular levels [1].

Cancer treatment in childhood can lead to premature onset of typically age‐related conditions including diabetes, metabolic syndrome, and cardiovascular disease, as well as secondary malignancies, neurocognitive impairment, sarcopenia and osteopenia, and frailty [2]. This suite of poly‐morbidities has been linked with senescent changes in the immune system in elderly individuals, but it is unknown whether these same conditions in childhood cancer survivors (CCS) are similarly underpinned by immunosenescence. Here, we compared peripheral blood immune cells and cytokines/chemokines from two cohorts of neuroblastoma CCS (nCCS) (survivors at 1–4 years, and at 5 or more years since diagnosis), healthy children, and elderly patients showing an immunosenescent phenotype (cohort characteristics shown in Supporting Information Table S1) in order to understand the effects of neuroblastoma treatment and to screen for signs of immune senescence.

Immunosenescence is recognized by increased plasma IL‐6, TNF‐α, and CRP [3] combined with expansion of CD8^+^ T cell subsets expressing CD57, a marker of proliferative senescence, and lacking CD28 and CD27 [4]. We first confirmed that our elderly cohort exhibited immunosenescent changes in T cell subsets relative to young controls, using flow cytometry to identify CD8^+^ and CD4^+^ subpopulations based on differential expression of CD45RA, CD45RO, CD27, and CD57 (gating strategy shown in Supporting Information Figure S1; subset comparison shown in Supporting Information Table S2). We then examined T cell subsets in the blood of nCCS and found that survivors at 1–4 years post‐diagnosis had significantly lower frequencies of naïve CD8^+^ T cells (CD3^+^CD8^+^CD45RA^+^CD45RO^−^CD27^+^) and significantly higher frequencies of CD8^+^ memory T cells expressing CD57 (CD3^+^CD8^+^CD45RA^−^CD45RO^+^CD57^+^) within their CD8^+^ populations, and in absolute terms, compared to healthy young controls (Fig. 1A). However, by 5 or more years after diagnosis, these levels had normalized (Fig. 1A). In contrast, frequencies and numbers of CD57^+^ terminally differentiated effector memory cells re‐expressing CD45RA (TEMRA, CD45RA^+^CD45RO^−^CD27^−^CD57^+^) do not differ between groups (Supporting Information Table S3).

**Figure 1:**
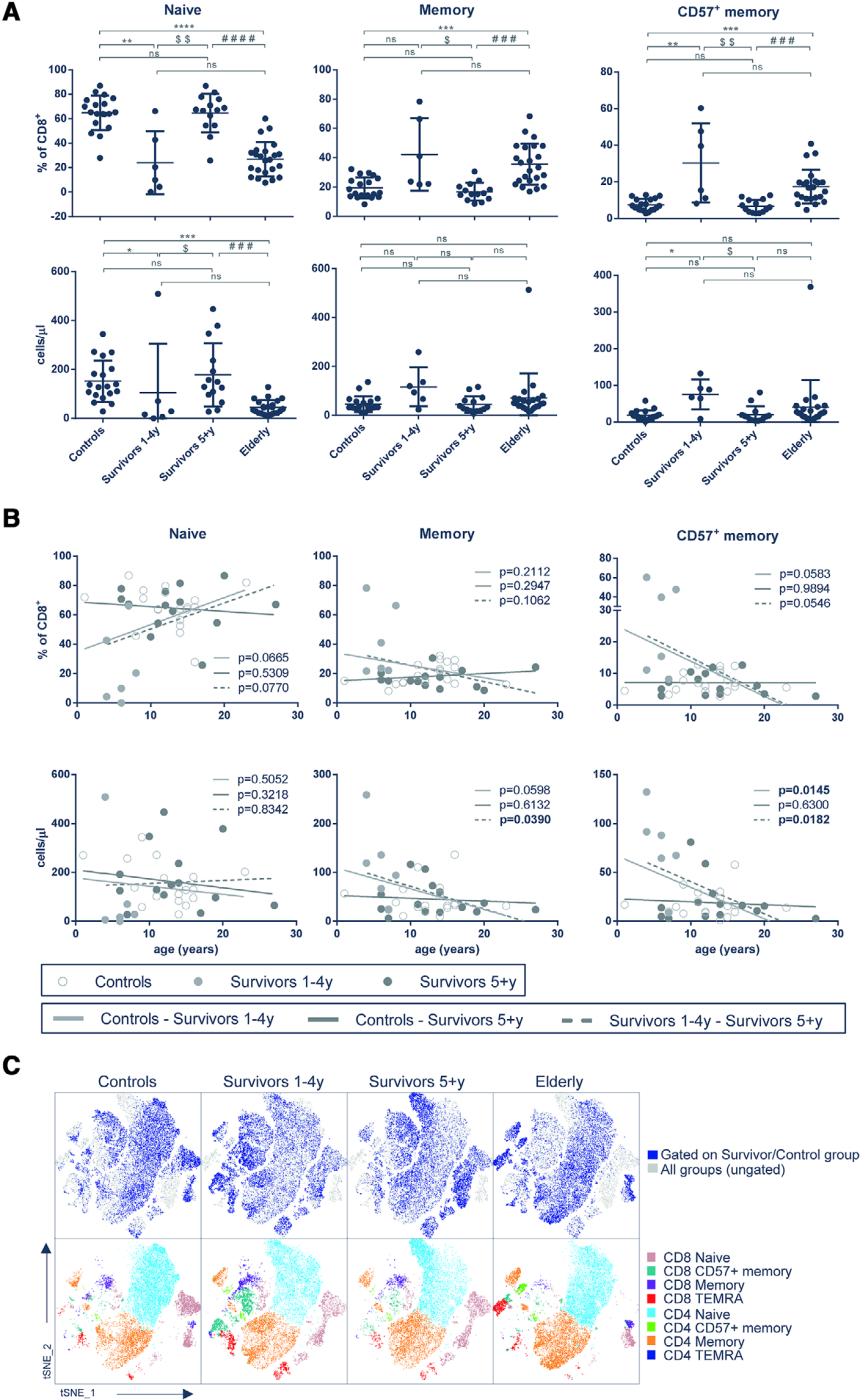
**Changes in relative abundance of CD8^+^ T cell subsets**. (**A**) Frequencies of naïve (CD45RA^+^CD45RO^−^CD27^+^), memory (CD45RA^−^CD45RO^+^), and memory expressing CD57 (CD45RA^−^CD45RO^+^CD57^+^) cells in the CD3^+^CD8^+^ compartment are shown and were measured by flow cytometry. Data are presented as mean ± SD. Individual groups were compared using the Kruskal–Wallis test followed by Dunn's multiple comparison test. Symbol indicates statistically significant difference in comparison to young healthy Control cohort (*), Elderly (MCI cohort) (#), and between Survivors 1–4 years and Survivors 5+ years ($). Number of symbols corresponds to *p*‐value level (one *p* < 0.05, two *p* < 0.01, three *p* < 0.001, four *p* < 0.0001, ns – not significant). **(B)** Linear regression analysis of the percentage and absolute CD8^+^ T cell number per μl of whole blood with age. Linear regression was performed for following group pairs, Controls – Survivors 1–4 years (light grey line), Controls – Survivors 5+ years (dark grey line) and Survivors 1–4 – Survivors 5+ years (dashed line) and the *p*‐value for each pair is shown. For (A) and (B), the number of samples per group is as follows: Controls *n* = 19, Survivors 1–4 years (*n* = 6), Survivors 5+ years (*n* = 14) and Elderly (*n* = 23) and data were acquired individually at the day of sample collection. **(C)** T cell subset distribution after dimensionality reduction analysis with t‐SNE. The upper panel shows distribution of CD3^+^ cells in individual groups (blue) within CD3^+^ cells of all groups (grey). The lower panel depicts specific CD8^+^ and CD4^+^ T cell subsets in individual groups. Four representative samples per group are included in the tSNE analysis.

Analyzing these data further using linear regression revealed differences in the relative frequency and absolute number of CD8^+^ T cell memory subsets with age (Fig. 1B), confirming the immuno‐phenotypic changes in the Survivor 1–4 years group. We also uncovered a significantly lower naïve/memory CD8^+^ T cell ratio in the Survivors 1–4 years group compared to young controls (Supporting Information Table S3), and a significantly higher total lymphocyte count (Supporting Information Table S4). CD4^+^ T cell subsets were not markedly affected by neuroblastoma treatment (Supporting Information Fig. S2A and B). Taken together, nCCS at early time‐points post‐treatment show significant differences in their CD8^+^ T cell compartment compared to children who have not undergone cancer treatment, but by 5 or more years post‐diagnosis the lymphocyte subsets of nCCS resemble those of healthy control children.

To understand whether the nCCS 1–4 years group were exhibiting an immunosenescent T cell profile, we compared them with elderly controls. We found similar trends in CD8^+^ and CD4^+^ T cell subsets in nCCS and elderly groups, but these trends had reverted to a normal phenotype by 5+ years after neuroblastoma diagnosis (Fig. 1A; Supporting Information Fig. S2A). We visualized the global differences in T cell subsets in each group using tSNE dimensionality reduction, which clearly showed the unique profile of nCCS at 1–4 years post‐diagnosis, the similarities between young controls and nCCS at 5 or more years post‐diagnosis, and the distinct immunosenescent profile present in elderly controls (Fig. 1C).

Aside from alterations in T cell subsets, chronic low‐grade inflammation (CLGI) is a common consequence of cancer treatment and a characteristic feature of immune‐senescence in the elderly [5]. We therefore measured the plasma levels of cytokines associated with CLGI and immune‐senescence in our cohorts. We found a significantly higher concentration of the pro‐inflammatory cytokine TNF‐α in the plasma of Survivors 1–4 years in comparison to age‐matched controls (Supporting Information Table S5). Similarly, the elderly group expressed higher levels of TNF‐α and other cytokines related to the senescence‐associated secretory phenotype [6, 7], while exhibiting lower levels of the anti‐inflammatory cytokine IL‐4 and markers associated with age‐related pathologies (Supporting Information Table S5).

Overall, our data show that nCCS in the first years of survivorship exhibit variable but significant changes in the relative frequencies of CD8^+^ T cell subsets compared to healthy children, accompanied by higher concentrations of TNF‐α in plasma, as seen in elderly controls with immunosenescence‐related CLGI. Importantly, and unexpectedly, from 5 years after diagnosis and treatment we see signs of immune reconstitution, indicating either that the immune system is able to recover from the toxicity of treatment, or that short‐term and long‐term adverse effects are separated by a period of apparent immune‐normality. Although the early dysregulation of the immune system in nCCS phenocopies some aspects of immunosenescence, this is likely not a result of premature aging of the immune system but rather the direct effects of neuroblastoma therapy. These findings are encouraging for the medium‐term prognosis of nCCS, but do not exclude the possibility of premature immune aging at later time points than studied here, as reported in childhood leukemia survivors [8, 9]. The immune parameters and panels presented in this study form a useful basis for future studies in older CCS, especially for identification and monitoring of those individuals with prolonged sub‐optimal immune phenotypes and thus potentially higher risk of adverse health consequences.

## Conflict of Interest

The authors declare no commercial or financial conflict of interest.

**Abbreviations**
CCS: childhood cancer survivors
CLGI: chronic low‐grade inflammation
HSCT: hematopoietic stem cell transplantation
nCCS: neuroblastoma childhood cancer survivors
TEMRA: terminally differentiated effector memory T cells re‐expressing CD45RA

## Supporting information

Supporting information
