# Supplementary material for: Childhood survivors of high‐risk neuroblastoma show signs of immune recovery and not immunosenescence
Source: Eur J Immunol. 2020 Aug 18;50(12):2092–4. doi: 10.1002/eji.202048541 (PMC7754117; doi:10.1002/eji.202048541)
Supplement: Supplementary file 1 — Supporting information [file EJI-50-2092-s001.pdf]

Supporting Information

Childhood survivors of high-risk neuroblastoma show signs of immune recovery and not immunosenescence.

Petra Lázničková,[1,2] Tomáš Kepák,[1,3] Marcela Hortová – Kohoutková,[1] Luděk Horváth,[1] Kateřina Sheardová,[1,4] Rafal Marciniak,[1] Carmine Vacca,[5] Michaela Šiklová,[6] Teresa Zelante,[5] Lenka Rossmeislová,[6] Zdenka Křenová,[3] Jaroslav Štěrba,[1,3] Kamila Bendíčková[1] and Jan Frič[1,7]

[1]International Clinical Research Center, St. Anne's University Hospital Brno, Brno, Czech Republic.

[2]Department of Biology, Faculty of Medicine, Masaryk University, Brno, Czech Republic.

[3]Department of Paediatric Oncology, University Hospital Brno, Brno, Masaryk University, Czech Republic.

[4]1st Neurology Department, St. Anne's University Hospital Brno, Brno, Czech Republic.

[5]Department of Experimental Medicine, University of Perugia, Perugia, Italy.

[6]Department of Pathophysiology, Third Faculty of Medicine, Charles University, Prague, Czech Republic.

[7]Institute of Hematology and Blood Transfusion, Prague, Czech Republic

Supporting Information

Supporting data

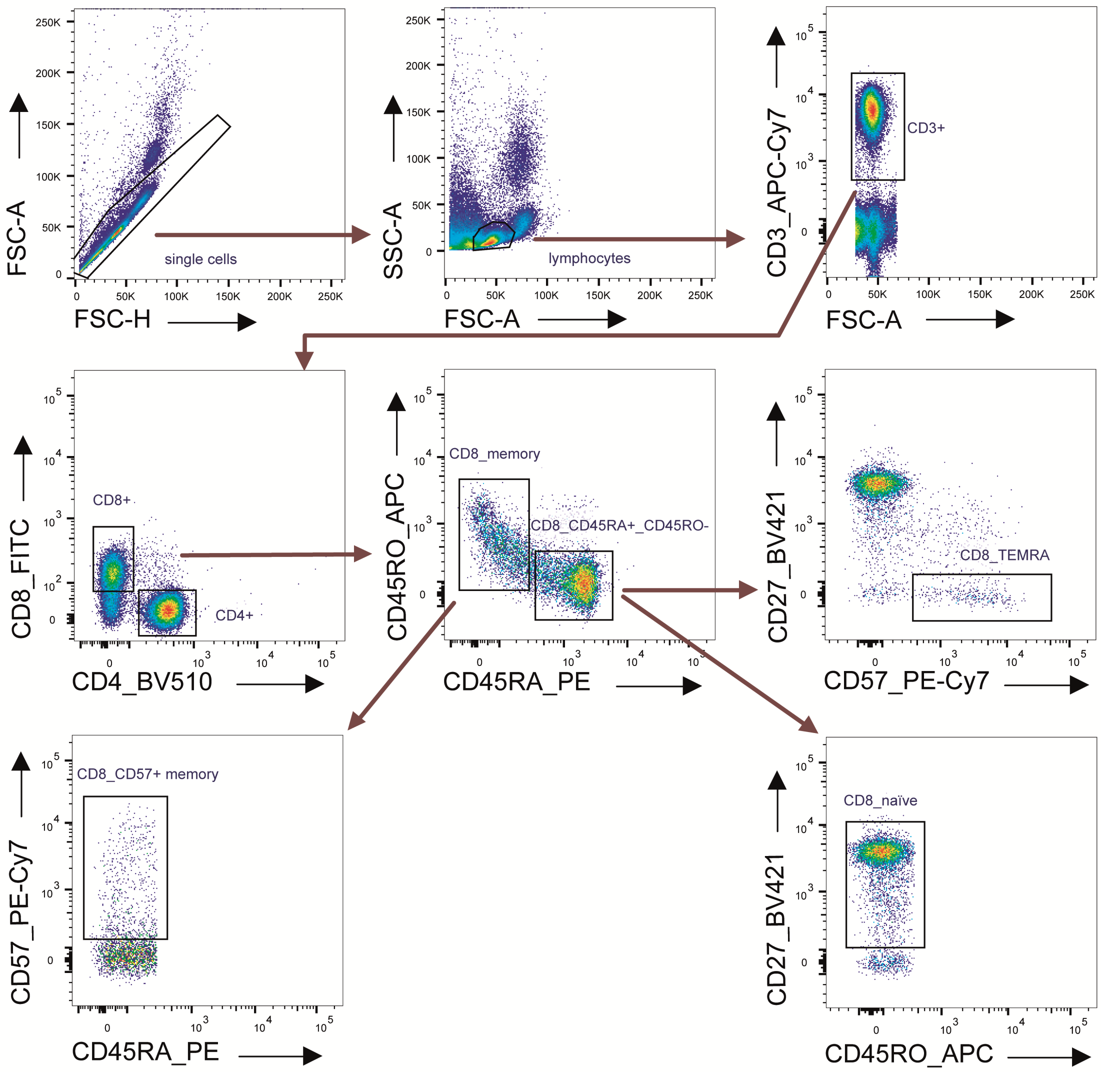

Supplementary Figure 1: Gating strategy used for T cell phenotyping. We first gated on $CD3^+CD4^+$ and $CD3^+CD8^+$ T cell populations and then divided each into $CD45RA^-CD45RO^+$ (memory T cell) and $CD45RA^+CD45RO^-$ sub-populations. Within the $CD45RA^+CD45RO^-$ T cell population we used expression of the proliferative marker of senescence CD57 and the stimulatory molecule CD27 to delineate sub-populations of naïve ($CD45RA^+CD45RO^-CD27^+$) and terminally differentiated effector memory cells re-expressing CD45RA (TEMRA)($CD45RA^+CD45RO^-CD27^-CD57^+$). We also identified memory T cells expressing CD57 ($CD45RA^-CD45RO^+CD57^+$). Only dot-plots for $CD8^+$ T cells are shown; the same gating strategy was used to distinguish $CD4^+$ subsets.

Supporting Information

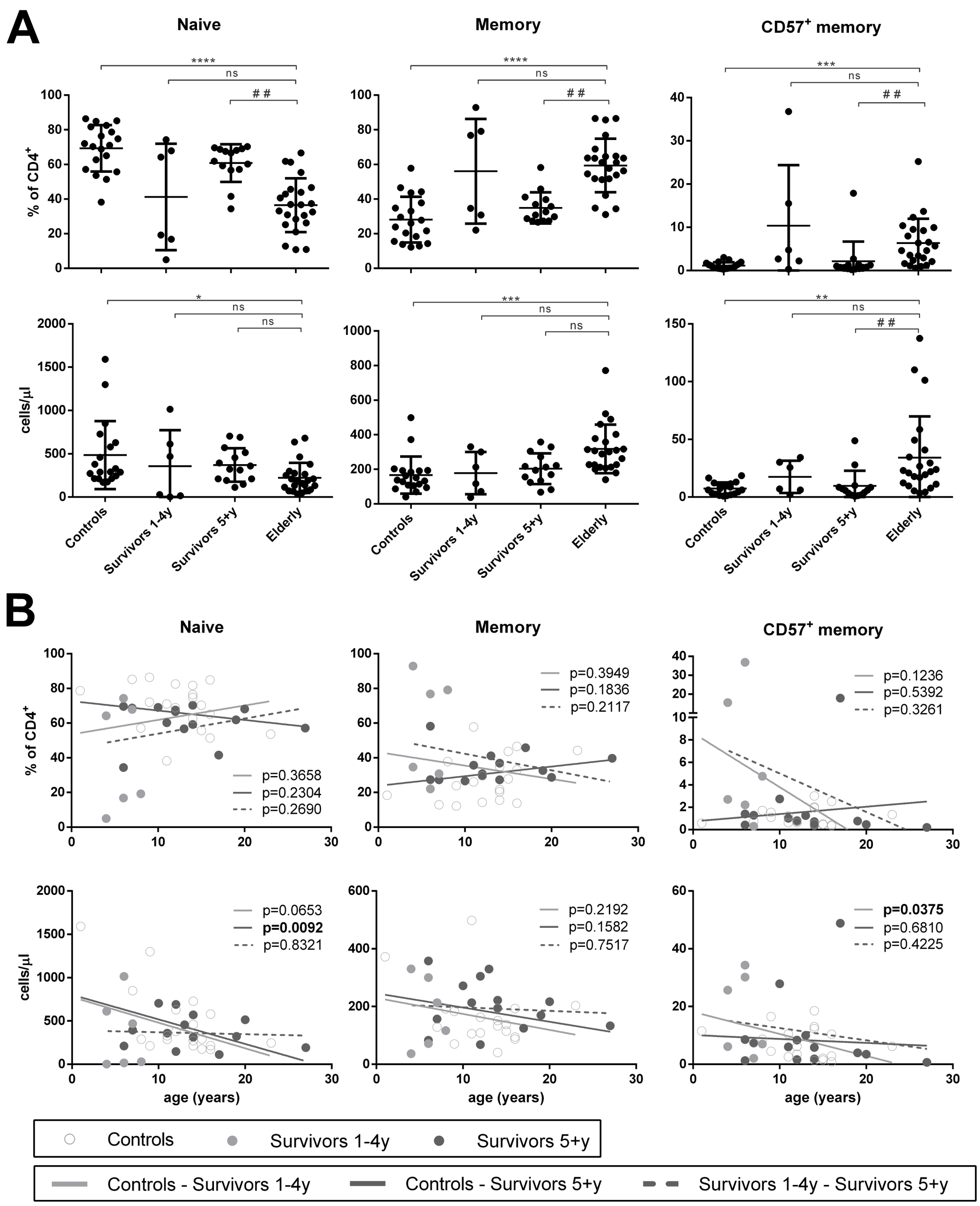

Supplementary Figure 2: (A) Changes in relative abundance of CD4$^+$ T cell subsets. Frequencies of naïve (CD45RA$^+$CD45RO$^-$CD27$^+$), memory (CD45RA$^-$CD45RO$^+$), memory expressing CD57 (CD45RA$^-$CD45RO$^+$CD57$^+$) cells in CD3$^+$CD4$^+$ compartment are shown. Data are presented as mean ± SD. Individual groups were compared using the Kruskal-Wallis test followed by Dunn's multiple comparison test. Symbol indicates statistically significant difference in comparison to young healthy Control cohort (*) and Elderly (MCI cohort) (#). Number of symbols corresponds to p-value level (one – $p < 0.05$, two – $p < 0.01$, three – $p < 0.001$, four – $p < 0.0001$, ns – not significant). **(B)** Linear regression analysis of proportion and absolute CD4$^+$ T cell number per µl of whole blood with age. Linear regression was performed for following group pairs, Controls – Survivors 1-4y (light grey line), Controls – Survivors 5+y (dark grey line) and Survivors 1-4y – Survivors 5+y (dashed line), the p value for each pair is shown. Significant p-values ($p<0.05$) are written in bold. For (A) and (B), the number of samples per group is as follows – Controls n=19, Survivors 1-4y (n=6), Survivors 5+y (n=14) and Elderly (n=23).

Supporting Information

Tables

Supplementary Table 1: Demographic and clinical characteristics of cohorts.

**nCCS**	**Survivors 1-4y**	**Survivors 5+y**	**p value**	
Number of participants (n)	14	22	–	
Sex (male/female), n (%)	9 (64)/5 (36)	13 (59)/9 (41)	>0,9999	
Age at study recruitment, median (min-max)	5 (2-8)	12 (5-27)	<0,0001	
Age at diagnosis - median (min-max)	2 (0-7)	2 (0-12)	0.824	
Years after transplantation, median (min-max)	2 (0-3)	8 (2-18)	<0,0001	
Years since diagnosis, median (min-max)	3 (1-4)	8 (5-20)	<0,0001	
Autologous HSCT, n (%)	14 (100)	22 (100)	>0,9999	
Chemotherapy, n (%)	14 (100)	22 (100)	>0,9999	
Radiotherapy, n (%)	13 (93)	22 (100)	>0,9999	
Relapse, n (%)	2 (14)	2 (9)	0.6092	
Death, n (%)	1 (7)	0 (0)	0.3611	
Refractory disease, n (%)	2 (14)	3 (14)	>0,9999	
**Child controls**			**p value (Survivors 1-4y)**	**p value (Survivors 5+y)**
Number of participants (n)	19		–	–
Sex (male/female), n (%)	6 (32)/13 (68)		0.8382	0.4971
Age at study recruitment, median (min-max)	14 (1-23)		0.2613	>0,9999
**Elderly – Mild Cognitive Impairment (MCI)**			**p value (Survivors 1-4y)**	**p value (Survivors 5+y)**
Number of participants (n)	23		–	–
Sex (male/female), n (%)	8 (35)/15 (65)		>0,9999	0.6613
Age at study recruitment, median (min-max)	74 (57-71)		<0,0001	<0,0001
MMSE mean, SD (min-max)	27.26, 1.63 (24-29)		–	–
**Elderly (EXODYA)**			**p value (Survivors 1-4y)**	**p value (Survivors 5+y)**
Number of participants (n)	23		–	–
Sex (male/female), n (%)	0 (0)/ 23 (100)		0.0023	0.0002
Age at study recruitment, median (min-max)	72 (61-79)		< 0,0001	< 0,0001
BMI, median (min-max)	27 (22-36)		–	–

P value was calculated using the Kruskal Wallis test followed by Dunn's multiple comparison.

Supporting Information

Supplementary Table 2: T-cell subset distribution in young healthy controls versus elderly (MCI).

		Controls	Elderly (MCI)	Proportional/ absolute cell count differences (p value)	Age-related differences (p value)
$CD8^+$ naive	(%)	65.30 (27.90-86.90)	26.60 (7.69-60.10)	< 0.0001	< 0.0001
	(cells/µl)	130 (28-344)	39 (10-128)	0.0001	< 0.0001
$CD8^+$ memory	(%)	16.00 (8.20-32.20)	33.40 (16.90-68.30)	0.0012	< 0.0001
	(cells/µl)	33 (10-136)	49 (17-514)	> 0.9999	0.3249
$CD8^+$ $CD57^+$ memory	(%)	6.89 (2.74-12.80)	16.70 (4.61-40.80)	0.0005	< 0.0001
	(cells/µl)	16 (1-58)	24 (5-368)	0.6242	0.2320
$CD8^+$ TEMRA	(%)	3.17 (0.80-34.00)	27.00 (5.10-49.00)	0.0001	< 0.0001
	(cells/µl)	7 (1-159)	37 (3-295)	0.0193	0.1302
$CD4^+$ naive	(%)	71.00 (38.20-86.40)	36.70 (10.80-66.60)	< 0.0001	< 0.0001
	(cells/µl)	291 (172-1591)	180 (36-682)	0.0129	0.0011
$CD4^+$ memory	(%)	26.00 (12.20-57.80)	61.10 (31.00-86,50)	< 0.0001	< 0.0001
	(cells/µl)	138 (41-498)	301 (140-771)	0.0001	0.0009
$CD4^+$ $CD57^+$ memory	(%)	0.81 (0.34-3.00)	4.63 (0.58-25.20)	0.0003	< 0.0001
	(cells/µl)	6 (1-19)	22 (4-137)	0.0012	0.0023
$CD4^+$ TEMRA	(%)	0.02 (0.00-0.31)	0.27 (0.00-7.32)	0.0009	0.0172
	(cells/µl)	0 (0-5)	2 (0-21)	0.0009	0.0089

Data represented as median (min-max). P value for proportional/absolute cell count differences was calculated using the Kruskal Wallis test followed by Dunn's multiple comparison, p value for age-related differences was calculated from linear regression analysis.

Supporting Information

Supplementary Table 3: Relative and absolute abundance of $CD4^+$ and $CD8^+$ T cell subsets within $CD3^+$ cell population in peripheral blood of young healthy controls, nCCS groups and elderly (MCI).

	**Controls**	**Survivors 1-4y**	**Survivors 5+y**	**Elderly (MCI)**
$CD4^+$ (% of $CD3^+$)	61.59 ± 10.2	42.57 ± 19.8$^{\#}$	55.52 ± 14.4	67.42 ± 11.8
$CD8^+$ (% of $CD3^+$)	23.62 ± 8.4	39.95 ± 20.0	24.11 ± 9.5	22.26 ± 10.7
$CD4^+$ TEMRA (% of $CD3^+CD4^+$)	00.06 ± 0.09	00.43 ± 0.8	00.41 ± 1.1$^{\#}$	01.06 ± 1.7***
$CD4^+$ TEMRA (cells/µl)	0.52± 1.1	1.40± 2.6	1.92± 4.7	4.34± 5.9
$CD8^+$ TEMRA (% of $CD3^+CD8^+$)	08.72 ± 10.5	15.78 ± 17.1	10.67 ± 12.7$^{\#\#}$	27.05 ± 12.6***
$CD8^+$ TEMRA (cells/µl)	27.04 ± 41.4	74.95 ± 136	33.18 ± 69.6	55.13 ± 59.8*
$CD4^+$/ $CD8^+$ ratio	03.18 ± 1.9	01.93 ± 2.4$^{\#}$	02.94 ± 1.9	03.88 ± 2.2
$CD4^+$ naïve/memory ratio	03.29± 2.0	01.33± 1.4	01.90 ± 0.6$^{\#\#}$	00.74 ± 0.5****
$CD8^+$ naïve/memory ratio	04.00 ± 2.2	0.85 ± 1.2**	04.49 ± 2.4$^{\#\#\#\#/\$\$\$}$	00.95 ± 0.7****

Data represented as (mean ± SD). Individual groups were compared using the Kruskal-Wallis test followed by Dunn's multiple comparison test. Symbol indicates statistically significant difference in comparison to Control cohort (*), Elderly (MCI cohort) (#), and between Survivor 1-4y and Survivor 5+y ($). Number of symbols corresponds to p value level (one – $p < 0.05$, two – $p < 0.01$, three – $p < 0.001$, four – $p < 0.0001$). TEMRA - terminally differentiated effector memory cells re-expressing CD45RA ($CD45RA^+CD45RO^-CD27^-CD57^+$). Ratios were calculated from the relative proportions of $CD4^+$ and $CD8^+$ or from respective naïve and memory subsets within the $CD3^+$ population.

Supporting Information

Supplementary Table 4: White blood cell counts in nCCS and young healthy controls.

		Controls	Survivors 1-4y	Survivors 5+y
WBC	$x10^9/L$	6.371±1.43	7.078±2.44	6.960±2.26
Neutrophils	$x10^9/L$	3.364±1.38	3.157±2.34	3.672±2.06
	% of WBC	51.7±12.3	40.4±14.6*	50.9±11.9
Lymphocytes	$x10^9/L$	2.231±0.76	3.112±0.98*	2.419±0.81
	% of WBC	35.8±11.4	45.9±15.0	36.2±10.0
Monocytes	$x10^9/L$	0.515±0.13	0.771±0.42	0.609±0.20
	% of WBC	8.2±1.8	10.5±3.4	9.0±2.6
Basophils	$x10^9/L$	0.029±0.02	0.04±0.029	0.039±0.03
	% of WBC	0.5±0.3	0.7±0.7	0.5±0.4
Eosinophils	$x10^9/L$	0.232±0.21	0.160±0.11	0.206±0.19
	% of WBC	3.6±3.1	2.2±1.3	3.1±2.6

Data represented as (mean ± SD). Individual groups were compared using the Kruskal Wallis test followed by Dunn's multiple comparison. * indicates statistically significant difference in comparison to Control cohort. Number of symbols corresponds to p value level (one – $p < 0.05$).

Supplementary Table 5: Molecules quantified in plasma samples from nCCS patients,young healthy controls and elderly (MCI, EXODYA).

		Controls	Survivors 1-4y	Survivors 5+y	Elderly MCI+EXODYA	Function associated with
CRP (mg/L)	Median	0.549	1.731	2.293	1.285^{A}	Inflammation and aging related diseases [1]
	min-max	0–6.823	0–6.452	0.202–6.03	0–6.227	
IL-6 (pg/mL)	Median	1.224	0.9315	1.164	1.784^{A}	SASP [2]
	min-max	0.168–4.186	0.138–5.843	0.505–3.318	0.333–6.182	
TNF-α (pg/mL)	Median	0.676	*1.139	0.8575	*1.084^{A}	SASP, inflammation [3-5]
	min-max	0.115–2.797	0.301–2.503	0.087–1.884	0.57–1.922	
BDNF (pg/mL)	Median	74.82	64.73	31.02	*11.93^{A}	Alzheimer's disease[6, 7]
	min-max	45.83–135.7	11.17–232.1	11.94–100.2	4.365–73.96	
CCL-11 (pg/mL)	Median	17.68	##24.72	##25.84	12.44^{A}	Aging[8]
	min-max	12.27–38.31	14.58–58.32	15.45–63.4	4.49–19.86	
CXCL-1 (pg/mL)	Median	9.415	6.158	8.01	6.27^{A}	SASP [5]
	min-max	5.205–23.52	4.44–10.63	3.005–13.63	5.325–13.03	
HGF (pg/mL)	Median	33.96	63.8	51.41	63.48^{A}	SASP [5]
	min-max	16.57–158.8	25.98–168.5	27.02–77.04	31.08–115.3	
IFNα (pg/mL)	Median	1.655	1.958	2.21	2.255^{A}	Aging – decreased secretion with age [9]
	min-max	0.71–11.58	0.945–2.715	1.17–2.625	1.95–3.595	

Supporting Information

CXCL-10 (pg/mL)	Median	6.048	##7.83	####6.135	****14.25A	SASP[10]
	min-max	4.715–8.555	6.25–16.6	3.41–8.91	5.605–22.19	
CCL-2 (pg/mL)	Median	46.08	87.97	35.76	85.15A	SASP [5]
	min-max	19.6–85.03	44.09–236.5	16.64–185.7	20.39–120.5	
PDGF-BB (pg/mL)	Median	22.4	23.86	####18.46	**88.08A	SASP[11]
	min-max	16.95–36.05	19.93–37.9	16.95–45.11	35.08–543.8	
PIGF-1 (pg/mL)	Median	32.58	###36.61	28.48	*19.06A	Atherogenesis [12]
	min-max	17.99–42.86	29.8–48.69	18.76–45.94	6.885–30.48	
CCL-5 (pg/mL)	Median	105	127.1	84.5	131.1A	SASP [5]
	min-max	50.97–217.8	60.86–219.5	38.9–217.2	40.08–347.2	
SCF (pg/mL)	Median	12.35	10.56	#12.59	*4.478A	Hematopoiesis [13]
	min-max	3.8–32.37	3.525–20.77	3.15–18.22	2.32–6.815	
CXCL-12a (pg/mL)	Median	597.8	#603.8	542.4	434.2A	Wound healing [14]
	min-max	323.3–799	531.4–718.7	421.8–909.6	328–717.2	
VEGF-A (pg/mL)	Median	49.68	94.99	59.15	70.38A	SASP [5]
	min-max	34.26–247.5	44.84–186	33.45–395.3	38.26–199	
VEGF-D (pg/mL)	Median	9.435	7.565	11.76	10.28A	SASP [5]
	min-max	5.66–32.62	3.935–16.41	7.75–14.81	3.91–27.58	
IL-10 (pg/mL)	Median	8.63	6.47	7.615	7.27B	Anti-inflammatory response [15]
	min-max	2.89–21.1	4.05–12.23	3.12–12.91	4.29–15.69	
IL-13 (pg/mL)	Median	10.99	8.17	8.42	9.82B	SASP [5]
	min-max	3.4–21.83	3.73–19.28	6.09–19.1	1.34–20.99	
IL-17A (pg/mL)	Median	10.2	#6.935	9.75	10.32B	Inflammation [16]
	min-max	4.33–23.77	2.85–15.9	4.49–17.04	4.65–28.28	
IL-4 (pg/mL)	Median	39.9	###38.02	###34.21	****6.6B	Anti-inflammatory response [17]
	min-max	11.42–46.37	11.42–55.81	7.85–51.8	2.92–43.62	
IL-8 (pg/mL)	Median	5.54	##6.69	####5.42	****10.7B	SASP [10]
	min-max	2.92–9.3	4.02–10.75	1.72–7.64	7.15–24.78	

Individual groups were compared using the Kruskal-Wallis test followed by Dunn's multiple comparison test. Symbol indicates statistically significant difference in comparison to Control cohort (*) and Elderly (#). Number of symbols corresponds to p value level (one – $p < 0.05$, two – $p < 0.01$, three – $p < 0.001$, four – $p < 0.0001$).

CRP – C reactive protein, IL– interleukin, CCL - C-C Motif Chemokine Ligand, CXCL - Chemokine (C-X-C motif) ligand, TNFα – tumor necrosis factor alpha, BDNF - Brain-derived neurotrophic factor, HGF - Hepatocyte growth factor, IFNα – interferon alpha, PDGF-BB - Platelet-Derived Growth Factor-BB, PIGF-1 - Placenta Growth Factor-1, SCF – Stem cell factor, VEGF - Vascular endothelial growth factor

[A] refers to elderly – Mild Cognitive Impairment (MCI) cohort

[B] refers to elderly healthy women cohort (EXODYA)

Supporting Information

Materials and methods

Participants

Demographic and clinical characteristics of nCCS, healthy young controls and elderly cohorts are described in Table 1. Patients that were successfully treated for HRNB (nCCS), and a control cohort of age-matched healthy subjects consisting of patients' siblings and healthy volunteers were recruited by the Department of Paediatric Oncology, University Hospital Brno, Brno, Czech Republic. Survivors of HRNB were recruited 1-4 years and 5 or more years since diagnosis. High risk neuroblastoma was defined as age at diagnosis >18 months, with stage IV (metastatic) disease and/or unfavourable histology and tumour biology (N-myc amplification). The positive control group for immunosenescent cellular phenotype was participants of the Czech Brain Aging study performed at St. Anne's University Hospital in Brno, Czech Republic, who had been diagnosed with mild cognitive impairment (MCI), with additional inclusion criteria published elsewhere [18]. These patients were selected because the immuno-senescent changes that occur during aging lead to CLGI, which is associated with cognitive decline and neurodegeneration.[19] A second elderly cohort (EXODYA study, Charles University in Prague, Prague, Czech Republic) was included for the analysis of plasma proteins.

All procedures on human volunteers were in accordance with the 1964 Helsinki declaration and approved by ethics committees of the involved hospitals. Informed consent was obtained from all participants included in the study.

Treatment regimens

Patients were treated according HRNB protocols (COG A397, COG ANBL0532 – regimen A, COG ANBL02P1, COG ANBL12P1, COG ANBL 1531 over last 20 years) with initial diagnostic biopsy followed by intensive induction chemotherapy (Cisplatin, Etoposide, Vincristin, Cyclophosphamide, Doxorubicin +/- Topotecan), second-look surgery and adjuvant consolidation high-dose chemotherapy (Busulphan / Melphalan or Carboplatin / Melphalan / Etoposide) with autologous haematopoetic stem cell transplantation. Post-transplant maintenance treatment consisted of adjuvant radiotherapy and 13CRA (13 cis retinoic acid) therapy.

Immuno-phenotype of T cells and white blood cell counts.

Peripheral blood mononuclear cells (PBMCs) from all subjects were isolated from 10 ml of heparin-Li treated blood by gradient centrifugation using Lymphoprep (density 1.077 g/ml; STEMCELL technologies) following the manufacturer's recommendations.

We began by designing and testing a set of immunosenescence phenotyping protocols on PBMCs in our elderly control cohort. The T cell immunophenotype was established by labelling with anti-CD3-APC-Cy7, anti-CD8-FITC, anti-CD27-BV421, anti-CD45RA-PE, anti-CD45RO-APC, anti-CD57-PE-Cy7 (all Biolegend) and anti-CD4-BV510 (BD Biosciences). $CD3^+CD4^+$ and $CD3^+CD8^+$ T cells were further classified into naïve ($CD45RA^+CD45RO^-CD27^+$), memory

($CD45RA^-CD45RO^+$), memory expressing CD57 ($CD45RA^-CD45RO^+CD57^+$) and TEMRA expressing CD57 ($CD45RA^+CD45RO^-CD27^-CD57^+$) [20-22], CCR7 expression was not assessed. Changes in numbers of these T cell subsets have been previously associated with aging.[21, 22] We then expanded this analysis to the nCCS and healthy age-matched control groups. Sample acquisition was performed using FACS Canto II (BD Biosciences) and data were analyzed using FlowJo v.10 (Tree Star). $CD8^+$ and $CD4^+$ subset distribution with dimensionality reduction analysis with t-SNE was performed using FlowJo v.10 plugin. Peripheral blood from all subjects was used for peripheral leukocyte analysis. Control and survivor groups cell counts were analysed in certified clinical laboratory of The University Hospital Brno and the subsets' absolute counts were calculated from frequency of $CD3^+$ cells within total lymphocytes. Absolute cell counts for samples from the Elderly (MCI) were counted from the frequency of $CD3^+$ cells within PBMC counts.

Plasma analysis - Enzyme-linked immunosorbent assay (ELISA)

The commercial DuoSet ELISA kit (R&D Systems) was used for CRP detection in diluted plasma samples (1:7000), Quantikine HS ELISA Human TNFα Immunoassay and Quantikine HS ELISA Human IL-6 Immunoassay (both R&D Systems) were used to measure TNFα and IL-6 in undiluted plasma samples. ELISA assays were performed as recommended by the manufacturer. Data acquisition was performed using Multiskan GO Microplate Spectrophotometer (Thermo Scientific).

Plasma analysis - LUMINEX

Cytokine/Chemokine/Growth Factor Convenience 45-Plex Human ProcartaPlex Panel 1 (Invitrogen) was used to measure cytokines and growth factors in undiluted plasma samples. The measurement was performed as recommended by the manufacturer with overnight incubation of plasma samples with magnetic beads. Acquisition of samples used a Bio-Plex 200 Systems (Bio-Rad) and MAGPIX (Thermo Fisher). Further analysis of IL-4, IL-6, IL-8, IL-10, IL-13, IL-17A and TNF-α was performed in plasma using HSTCMAG-28SK-09 MILLIPLEX MAP Human High Sensitivity T Cell Panel (Merck). Sample acquisition was performed on MagPix machine. The cytokines with levels below detection limits are not reported.

Statistical analysis

Graphpad Prism software v.6 was used for statistical analysis. Data were tested for normal distribution and parametric or non-parametric statistical tests were applied as appropriate. Statistical tests used are specified in the figure/table legends.

Supplemental references:

1 **Tang, Y.et al.,** *Clin Exp Pharmacol Physiol* 2017. **44 Suppl 1**: 9-14.
2 **Coppe, J. P.et al.,** *Annu Rev Pathol* 2010. **5**: 99-118.
3 **Franceschi, C.et al.,** *Ann N Y Acad Sci* 2000. **908**: 244-254.
4 **Franceschi, C. and Campisi, J.,** *J Gerontol A Biol Sci Med Sci* 2014. **69 Suppl 1**: S4-9.

5 **Freund, A.et al.,** *Trends Mol Med* 2010. **16**: 238-246.
6 **Tapia-Arancibia, L.et al.,** *Brain Res Rev* 2008. **59**: 201-220.
7 **Erickson, K. I.et al.,** *J Neurosci* 2010. **30**: 5368-5375.
8 **Hoefer, J.et al.,** *Front Aging Neurosci* 2017. **9**: 402.
9 **Agrawal, A.,** *Gerontology* 2013. **59**: 421-426.
10 **Alam, I.et al.,** *J Transl Med* 2019. **17**: 272.
11 **Yu, Y. C.et al.,** *Sci Rep* 2013. **3**: 1675.
12 **Matsui, M.et al.,** *J Am Soc Nephrol* 2015. **26**: 2871-2881.
13 **Comazzetto, S.et al.,** *Cell Stem Cell* 2019. **24**: 477-486 e476.
14 **Loh, S. A.et al.,** *Plast Reconstr Surg* 2009. **123**: 65S-75S.
15 **Moore, K. W.et al.,** *Annu Rev Immunol* 2001. **19**: 683-765.
16 **Stout-Delgado, H. W.et al.,** *Cell Host Microbe* 2009. **6**: 446-456.
17 **Minciullo, P. L.et al.,** *Arch Immunol Ther Exp (Warsz)* 2016. **64**: 111-126.
18 **Marciniak, R.et al.,** *Clinical Interventions in Aging* 2020: in press.
19 **Lin, T.et al.,** *Front Aging Neurosci* 2018. **10**: 236.
20 **Verma, K.et al.,** *PLoS One* 2017. **12**: e0177405.
21 **Larbi, A. and Fulop, T.,** *Cytometry A* 2014. **85**: 25-35.
22 **Xu, W. and Larbi, A.,** *Int J Mol Sci* 2017. **18**.
